# SUZ12 Loss Amplifies the Ras/ERK Pathway by Activating Adenylate Cyclase 1 in NF1-Associated Neurofibromas

**DOI:** 10.3389/fonc.2021.738300

**Published:** 2021-10-06

**Authors:** Weijie Li, Chenhao Hu, Xingnan Zhang, Binbin Wang, Zhen Li, Miao Ling, Shengqiao Sun, Chao Guo, Dezhi Li, Song Liu

**Affiliations:** ^1^ Department of Injury and Repair, and Beijing Key Laboratory of Central Nervous System Injury, Beijing Neurosurgical Institute, Capital Medical University, Beijing, China; ^2^ U 1195, Institut national de la santé et de la recherche médicale (INSERM) and University Paris-Sud and University Paris Saclay, Le Kremlin-Bicêtre, France

**Keywords:** SUZ12, ADCY1, RAS, microdeletion, neurofibromas NF1, MPNST = malignant peripheral nerve sheath tumor

## Abstract

Patients with germline neurofibromatosis type 1 (NF1) microdeletions frequently exhibit hereditary syndromes such as cardiovascular anomalies and have an increased risk of malignant peripheral nerve sheath tumors (MPNSTs). This study aimed to identify the genes codeleted with SUZ12 that are related to MPNST. We used differential gene expression and enrichment analyses to analyze the SUZ12-mutant and SUZ12-wild-type gene expression profiles in the GSE118186 and GSE66743 datasets in Gene Expression Omnibus (GEO). PPI network analysis combined with MPNST patient survival analysis was used to identify ADCY1, which catalyzes the conversion of ATP to cAMP, as a key gene. Moreover, chromatin immunoprecipitation sequencing (ChIP-Seq) showed that the distribution of H3K27me3 in the ADCY1 promoter region and gene body was significantly reduced in SUZ12-mutant cells. To verify the role of ADCY1 in SUZ12 mutation, we used RNA interference and plasmid transfection to interfere with SUZ12 expression in plexiform neurofibroma (pNF) and MPNST cell lines and then treated the cells with forskolin, IBMX and H89. ERK phosphorylation was accelerated and prolonged after siRNA transfection, especially in ipNF05.5 cells, and the intensity and duration of ERK activation were reduced after SUZ12 overexpression. Importantly, the level of p-ERK was consistent with that of Rap1-GTP. Moreover, H89 completely blocked Rap1 activation and the changes in the p-ERK level after SUZ12 siRNA transfection. In conclusion, our findings suggested that SUZ12 loss potentiates the effects of NF1 mutations by amplifying Ras signaling through the ADCY1/cAMP/Rap1/ERK pathway and that SUZ12 may serve as a therapeutic and prognostic biomarker in NF1-associated neurofibromas.

## Introduction

Neurofibromatosis type 1 (NF1) is an autosomal dominant disorder that affects multiple organ systems and has a wide range of variable clinical manifestations. The NF1 gene, located on chromosome 17q11.2, encodes the neurofibromin protein, which functions as a tumor suppressor (1). The average global prevalence of NF1 is approximately 1 case per 3,000 individuals (2). The most representative tumors are peripheral nerve sheath tumors, which include dermal or plexiform neurofibromas (pNFs) (3). In contrast to dermal neurofibromas, pNFs can progress to malignant peripheral nerve sheath tumors (MPNSTs), which are the leading cause of death in NF1 patients (4).

Most patients with NF1 have a small mutation in the NF1 gene (point mutation, small deletion, intragenic insertion or duplication). However, an estimated 4.7-11% of NF1 patients have large deletions encompassing the entire NF1 gene and its flanking regions (generally termed ‘NF1 microdeletions’) at 17q11.2 (5, 6), which are frequently associated with a severe clinical presentation. Most patients with microdeletion harbor a 1.4-Mb germline microdeletion encompassing the entire NF1 gene, 14 protein-coding genes and four microRNA genes (7). Genotype-phenotype analysis has shown that patients with microdeletion of the NF1 gene usually exhibit more severe clinical phenotypes than patients with intragenic NF1 mutations; the former are usually characterized by facial deformities and severe developmental delays, and NF1 microdeletion is associated with a high tumor burden and cardiovascular abnormalities (8). In addition, studies have shown that patients with NF1 gene microdeletion have an increased risk of MPNSTs. Among all NF1 patients, the lifetime risk of MPNST is estimated to be 8-13% (9). However, individuals with NF1 microdeletion have a lifetime risk of MPNST as high as 16-26%. In addition, MPNSTs may occur earlier in patients with NF1 microdeletion than in patients with intragenic NF1 mutations (10).

The increased risk of MPNST in patients with NF1 microdeletion may be related to hemizygosity of the suppressor of zeste 12 homolog (SUZ12) gene, which is located in the NF1 microdeletion region. Polycomb repressive complex 2 (PRC2), which contains several essential subunits—embryonic ectoderm development (EED), SUZ12, and retinoblastoma-binding protein 4/7 (RBBP4/7)—as well as two paralogous enzymatic subunits, i.e., enhancer of zeste homolog 1 and 2 (EZH1 and EZH2), plays a crucial role in gene silencing by establishing di- and trimethylation of histone H3 lysine 27 (H3K27me2 and H3K27me3, respectively) (11, 12). PRC2 structure is composed of two primary lobes, the catalytic lobe and the regulatory lobe. The catalytic lobe contains multiple domains and motifs that are involved in the methylation process such as the SUZ12 VEFS domain. And both the EED and the SUZ12 subunit facilitate the stabilization of the complex and can bind the H3 N-terminal tail together with H3K27me3 (13).

As a core component of PRC2, SUZ12 frequently exhibits biallelic inactivation in MPNSTs, suggestive of a tumor suppressor function in this tumor type (14, 15). The mutation rate of SUZ12 was as high as 56.1%, with the highest mutation rate among the four subunits of PRC2 (16). More importantly, loss of PRC2 function and concomitant inactivation of the tumor suppressor genes NF1 and CDKN2A are considered to be the most significant diagnostic markers of MPNST in the revised 2016 WHO Classification of Tumors of the Central Nervous System (17, 18). Recent studies showed that SUZ12 upregulated the RAS pathway, although the phosphorylation level of ERK did not change significantly after either overexpression or knockout of SUZ12 (14). In this study, we revealed the role of SUZ12 and the mechanism by which it affects the RAS/ERK pathway in pNF and MPNST.

## Materials and Methods

### Sequencing Data

Gene Expression Omnibus (GEO, http://www.ncbi.nlm.nih.gov/geo) is a public functional genomics data repository of high-throughput gene expression, gene chip and microarray data. Two gene expression datasets (GSE118186 and GSE66743) were downloaded from GEO (18). GSE118186 contains SUZ12-wild-type (SUZ12-WT, 2 cases) and SUZ12-mutant (SUZ12-Mut, 2 cases) RNA-Seq data and H3K27me3-related chromatin immunoprecipitation sequencing (ChIP-Seq) data for ipNF05.5 cells. GSE66743 is a gene chip dataset containing data for 30 MPNST patients, including the survival times of the patients.

### Identification of DEGs

To identify differentially expressed genes (DEGs) between SUZ12-WT and SUZ12-Mut cells, we used Trim Galore and FastQC to process and optimize the raw data. The hg38.fa file was downloaded from the UCSC Genome Browser as the reference genome sequence. After the index was constructed with Salmon, quantitative gene expression analysis was carried out on 4 samples. With p adj ≤ 0.05 and Log|FC|≥2 as the screening criteria, DESeq2 was used to perform differential gene expression analysis on 2 samples of SUZ12-WT and 2 samples of SUZ12-Mut cells. The hg38.fa file was used as the reference genome sequence to construct an index with Bowtie to complete the ChIP-Seq gene alignment. SAMtools was used to convert the compared SAM files into BAM files and sort them. DeepTools was used to convert the BAM files to BW files to complete the visualization of transcription start sites (TSSs).

### KEGG Pathway Enrichment Analysis, GO Enrichment Analysis and GSEA of DEGs

To understand the functions of the DEGs, we used R software to perform Gene Ontology (GO) enrichment analysis, Kyoto Encyclopedia of Genes and Genomes (KEGG) enrichment analysis and gene set enrichment analysis (GSEA). Set H (hallmark gene sets; 50 gene sets), a predefined GSEA dataset, was used in this study. The GO enrichment analysis results show the top 10 pathways in descending order, the KEGG enrichment analysis results show the top 20 pathways in descending order, and the GSEA results show activated and inhibited pathways.

### Protein-Protein Interaction Network Construction and Hub Gene Selection

The DEGs were uploaded to STRING (https://string-db.org/) to construct the PPI network. According to the network’s maximal clique centrality (MCC, associated median value), CytoHubba in Cytoscape was used to identify the top 10 core genes in the network and annotate the functions of the core genes.

### Cox Survival Analysis by Hub Gene Expression *via* Cox Proportional Hazards Regression

A total of 28 MPNST patients in GSE66743 were included in the survival analysis, and 2 MPNST patients with unknown survival times were removed. The patients were divided into high-risk groups and low-risk groups based on the average core gene expression levels. To analyze the relationships between the hub genes and survival, Cox proportional hazards regression was used for survival analysis with R software. Genes with P<0.05 were defined as key genes.

### Distribution of H3K27me3 in the Promoter Regions of Key Genes and Across the Genome

The gene bodies and promoter regions of key genes were searched in the NCBI database, and the BW file obtained by ChIP-Seq analysis was imported into Integrative Genomics Viewer (IGV). The H3K27me3 distribution in key gene promoter regions and across the genome were compared between SUZ12-WT and SUZ12-Mut cells.

### Tissue Specimens

Eight MPNST specimens were obtained during surgical resection from patients at Beijing Tiantan Hospital between 2019 and 2021. Two of these cases were sporadic MPNSTs, and six were NF1-associated MPNSTs. The histopathological diagnosis was assessed according to the 2016 WHO Classification of Tumors of the Central Nervous System by two independent neuropathologists. Part of each specimen was snap frozen and stored in liquid nitrogen, and the remainder was fixed with 4% formalin for histopathological and immunohistochemical examination. All procedures were approved by the Institutional Review Board of the Beijing Tiantan Hospital Ethics Committee.

### Cell Culture and Reagents

The human MPNST cell line sNF96.2 and pNF cell line ipNF05.5 were purchased from the American Type Culture Collection (ATCC, USA). All cell lines were maintained in Dulbecco’s modified Eagle’s medium (DMEM, Gibco, USA) supplemented with 10% fetal bovine serum (FBS, Gibco, USA) and maintained at 37°C in 5% CO_2_. Forskolin, 3-isobutyl-1-methylxanthine (IBMX) and H89 were purchased from MedChem Express (MCE, USA). Cells were treated with 25 µM forskolin and 100 µM IBMX for 20 min; this treatment is designated F/I in the text and figures. The protein kinase A (PKA) inhibitor H89 was applied at 10 µM 20 min prior to treatment with F/I.

### IHC

For immunohistochemistry (IHC), sections were sequentially incubated with appropriate primary antibodies (specific for SUZ12, H3K27me3, ADCY1 and p-ERK) according to the corresponding instructions, incubated with the secondary antibody and diaminobenzidine (Zhongshan Bio Corp, China), counterstained with hematoxylin, and visualized under a light microscope.

### RNA Interference and Plasmid Transfection

SUZ12 and NC siRNA were chemically synthesized with the following sequences: NC antisense: 5′-ACGUGA CACGUUCGGAGAATT-3′, SUZ12 siRNA antisense 1: 5′-UAUUGGUGCUAUGAGAUUCCGTT-3′, antisense 2: 5′-UUCUAGUGGCAAGAGGUUUGGTT-3′, and antisense 3: 5′-UAGAUGAAGCAUGAAGUUUCGTT-3′ (Sangon Biotech, China). The recombinant plasmid SUZ12-pENTER was constructed by Vigene Bio, China. siRNAs and plasmids were transfected into cells at 70-80% confluence with Lipofectamine RNAiMAX or Lipofectamine 3000, respectively, according to the manufacturer’s instructions (Invitrogen, USA).

### RT-PCR Analysis

Total RNA was extracted with TRIzol (Life Technology, USA), and reverse transcription was performed using a high capacity reverse transcription kit (Thermo Fisher Scientific, USA) according to the manufacturer’s protocol. Real-time PCR (RT-PCR) was performed using Power SYBR Green Master Mix (Thermo Fisher Scientific, USA) according to the manufacturer’s instructions. RT-PCR data were analyzed by the 2-^ΔΔCt^ method. The following primer sequences were used: SUZ12 (F: 5′-CAA ACT GAA GCA AGA GAT GAC C-3′; R: 5′-GCT ATG GCA GAG TTT AAG ATG C-3′).

### Western Blot Analysis

Total cell lysates were prepared using RIPA lysis buffer supplemented with PMSF (Solarbio, China). The protein concentration was determined using a BCA protein assay kit (Biosharp, China). Proteins were resolved by SDS-PAGE and electrotransferred to PVDF membranes (Millipore, USA). Anti-ERK2 (sc-1647), anti-adenylate cyclase 1 (sc-365350), and anti-p-ERK (sc-7383) antibodies were purchased from Santa Cruz Biotechnology Inc. (Santa Cruz, USA). Anti-SUZ12 (D39F6) antibodies were purchased from Cell Signaling Technology (CST, USA). The Rap1 activation assay was performed with an Active Rap1 Detection Kit (Cell Signaling Technology, USA) according to the instructions.

### Statistical Analysis

Statistical methods used for bioinformatic analysis are detailed in each section of the experimental methods. Statistical analysis was performed using GraphPad Prism version 8. The results were presented as the mean ± standard deviation (SD) of independent triplicate experiments. Statistical differences between groups were assessed using a Student’s t-test analysis. *P<0.05 was considered statistically significant, and ***P<0.001 and ****P<0.0001 were considered highly significant.

## Results

### Identification of DEGs

**Figure 1:**
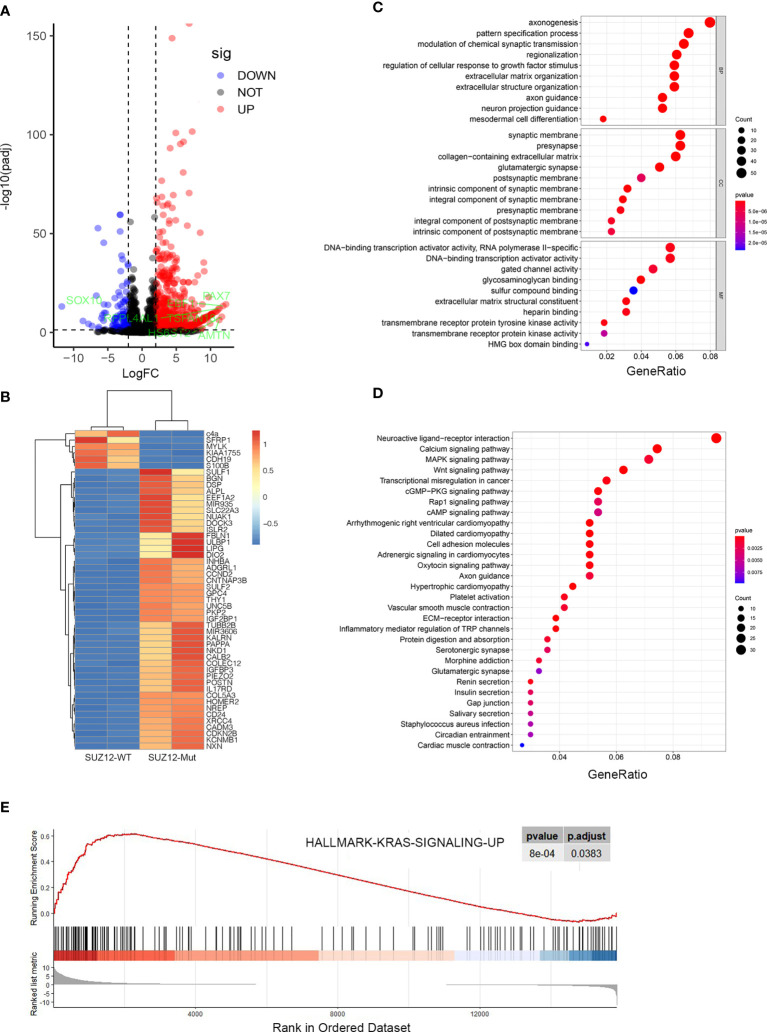
Identification and enrichment analysis of the differentially expressed genes in GSE118186 data. **(A)** Volcano maps of DEGs in the dataset was showed by using p adj ≤ 0.05 and Log|FC|≥2 as cut-off criteria. **(B)** Heatmap of top 50 differentially expressed genes in the dataset. **(C)** Gene Ontology (GO) enrichment analysis for the DEGs. **(D)** KEGG pathway enrichment analysis for the DEGs. **(E)** GSEA analysis showing DEGs enriched in KRAS pathway.

After normalization of the microarray results, DEGs were identified. A total of 704 genes were upregulated and 125 genes were downregulated in ipNF05.5 SUZ12-Mut cells compared with SUZ12-WT cells (**Figure 1A**). A heat map was constructed to show the names of top 50 genes (**Figure 1B**), and all DEGs were displayed in **Supplementary Table 1**.

### GO Enrichment Analysis, KEGG Enrichment Analysis and GSEA of the DEGs

To analyze the biological classification of the DEGs, functional and pathway enrichment analyses were performed using GO, KEGG and GSEA methods. GO analysis showed that in the DEGs were significantly enriched in the biological process (BP) terms axonogenesis, regulation of cellular response to growth factor stimulus and regionalization. In addition, the DEGs were enriched mainly in the cell component (CC) terms synaptic membrane, collagen-containing extracellular matrix, and glutamatergic synapse and in the molecular function (MF) terms DNA-binding transcription activator activity, glycosaminoglycan binding and extracellular matrix structural constituent (**Figure 1C**). Interestingly, KEGG pathway enrichment analysis revealed that the DEGs were enriched mainly in the cardiomyopathy and Wnt signaling pathways, and the gene ratios showed that more DEGs were clustered in the neuroactive ligand-receptor interaction pathway, MAPK signaling pathway, and Rap1 and cyclic adenosine monophosphate (cAMP) signaling pathway (**Figure 1D**). GSEA showed that the KRAS pathway was upregulated in SUZ12-Mut cells (**Figure 1E**).

### PPI Network Construction and Hub Gene Selection

**Figure 2:**
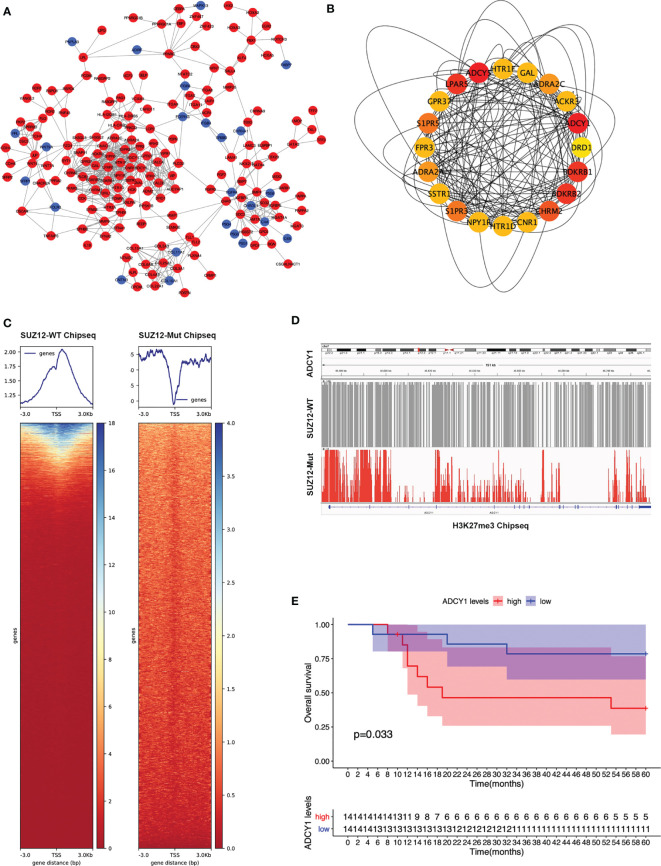
PPI network construction and the selection of hub genes. **(A)** The STRING database constructed the PPI network of DEGs. The red node represented the upregulated genes, and the blue node represented the downregulated genes. **(B)** The maximal clique centrality (MCC) algorithm was used to identify hub genes. The red node represented the gene with a high MCC score, while the yellow node represented the gene with a low MCC score. **(C)** Distribution of H3K27me3 in the TSS region of SUZ12-Mut and SUZ12-WT cells. The distribution of H3K27me3 was increased in SUZ12-WT cells and decreased in SUZ12-Mut cells. **(D)** The distribution of H3K27me3 in ADCY1 promoter region and genosome were compared and the result showed that it was significantly sparse in SUZ12-Mut cells. **(E)** Overall survival of hub gene ADCY1 was performed using COX survival analysis. P < 0.05 was considered statistically significant.

The PPI network of the DEGs was constructed (**Figure 2A**) and the most significant module identified using Cytoscape (**Figure 2B**). The top 10 hub genes were ADCY5, ADCY1, BDKRB1, LPAR5, BDKRB2, CHRM2, S1PR5, S1PR3, ADRA2A, and ADRA2C; all of these were upregulated DEGs. TSSs are one of the most important regions in the genome, and the altered distribution of H3K27me3 in TSS regions regulates gene expression. The distribution of H3K27me3 near TSS regions was significantly higher in SUZ12-WT cells than in SUZ12-Mut cells (**Figure 2C**). 4 of 10 hub genes were detected in the gene microarray dataset containing survival, and the other genes were not expressed. Subsequently, associations between overall survival and the expression levels of the 4 hub genes were analyzed using Cox proportional hazards regression for survival analysis (**Supplementary Figure S1**). MPNST patients with upregulation of only ADCY1 showed worse overall survival (**Figure 2E**). Importantly, compared with that in SUZ12-WT cells, the distribution of H3K27me3 in the ADCY1 promoter region and across the genome in SUZ12-Mut cells was sparse and significantly decreased (**Figure 2D**). KEGG pathway analysis showed that the Rap1 and cAMP signaling pathways were enriched, and the product of ADCY1 is cAMP. Therefore, it was speculated that SUZ12 deletion may amplify RAS signaling *via* the ADCY1/cAMP/Rap1/ERK pathway.

### Levels of SUZ12, H3K27me3, ADCY1 and p-ERK in MPNST Tumors

**Figure 3:**
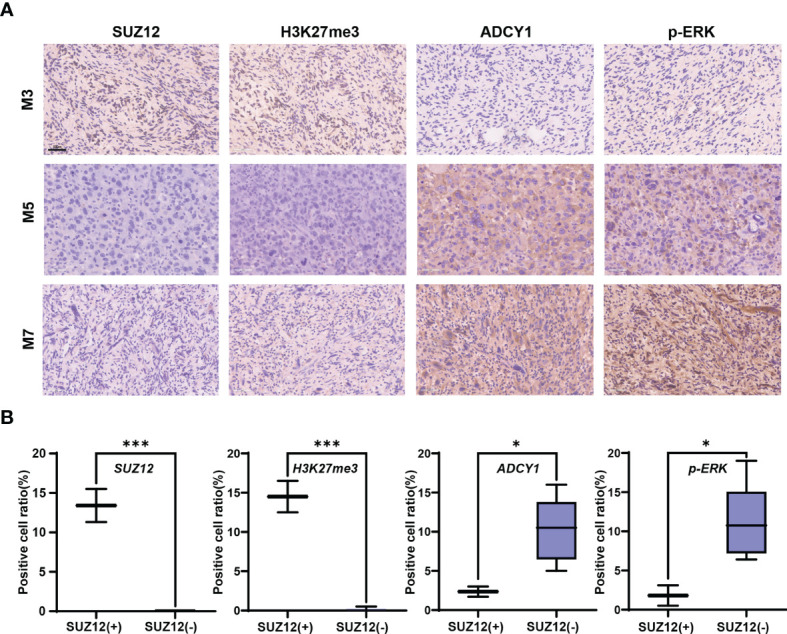
Expression of SUZ12, H3K37me3, ADCY1 and p-ERK in MPNST tumor samples. **(A)** The figure showing immunohistochemical results of SUZ12, H3K37me3, ADCY1 and p-ERK in tumor tissues of three MPNST patients. Scale bars: 50 µm (40 x). **(B)** The two groups were divided into SUZ12-positive (SUZ12+) and SUZ12-negative (SUZ12-) according to SUZ12 expression, and the positive cell ratio of SUZ12, H3K37me3, ADCY1 and p-ERK were compared between the two groups by t-test. ***P < 0.001, and *P < 0.05.

Clinical information and the SUZ12 expression data for 8 MPNST patients are shown in **Table 1**. Immunohistochemical analysis showed that two of the eight MPNST cases were positive for SUZ12, the remaining six did not express SUZ12, and all recurrent MPNSTs were SUZ12-negative. The H3K27me3 level was extremely low in SUZ12-negative patients, while the rates of ADCY1 and p-ERK positivity were significantly higher in SUZ12-negative patients than in SUZ12-positive patients (**Figures 3A, B**). These results suggested that the expression of ADCY1 and phosphorylation of ERK may be regulated by SUZ12, consistent with the differential gene expression analysis results.

**Table 1:** Clinical information and SUZ12 expression in 8 patients with MPNST.

Patient	Gender	Site	Recurrence	NF1	*SUZ12*
**M1**	F	neck	N	N	+
**M2**	M	trunk	Y	N	–
**M3**	F	neck	N	Y	+
**M4**	F	neck	Y	Y	–
**M5**	F	trunk	Y	Y	–
**M6**	M	thigh	N	Y	–
**M7**	M	thigh	N	Y	–
**M8**	M	thigh	Y	Y	–

M=malignant peripheral nerve sheath tumor, N=“no”, Y=“yes”, ”+” means SUZ12 positive in IHC.

### Activation of ADCY1 Affects the Temporal Characteristics of ERK Phosphorylation

**Figure 4:**
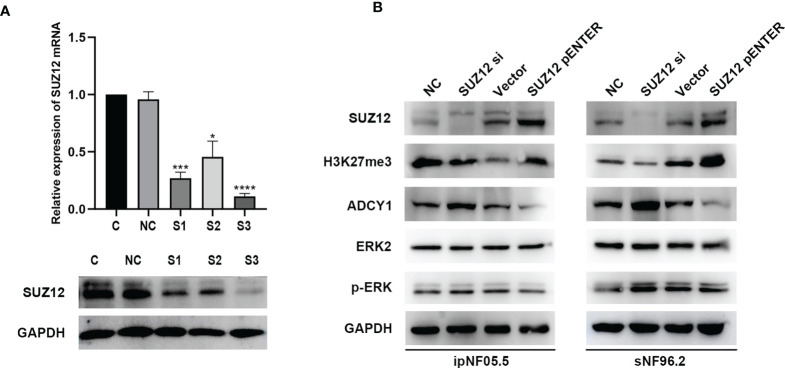
The expression of H3K27me3, ADCY1, ERK2 and p-ERK after SUZ12 was knocked down or upregulated. **(A)** Western blotting and RT-PCR showing that among the three siRNA sequences transfected, S3 was the most efficient (the statistical method was a t-test on the △△CT values, *P < 0.05, ***P < 0.001 and ****P < 0.0001). **(B)** Western blot showing SUZ12, H3K27me3, ADCY1, ERK2 and p-ERK levels in SUZ12 transfected interfering RNA (SUZ12 si) and plasmid groups (SUZ12 pENTER) in both ipNF05.5 and sNF96.2, and NC and vector as negative control respectively.

SUZ12 siRNA and pENTER-SUZ12 were transfected into ipNF05.5 and sNF96.2 cells to further clarify the effect of SUZ12, and NC siRNAs and empty vector were used as the corresponding controls. As shown by western blot analysis and RT-PCR, S3 had the best knockdown effect among the three siRNA sequences transfected and was thus used for all subsequent experiments (**Figure 4A**). The expression of SUZ12 was knocked down or upregulated after transfection of the siRNA and overexpression plasmid, respectively. In addition, after transfection, the H3K27me3 level was consistent with the SUZ12 expression level but was inversely related to the ADCY1 level. SUZ12 did not affect the expression level of ERK2, while the level of p-ERK varied, albeit non significantly, with that of ADCY1 in both ipNF05.5 and sNF96.2 cells (**Figure 4B**).

**Figure 5:**
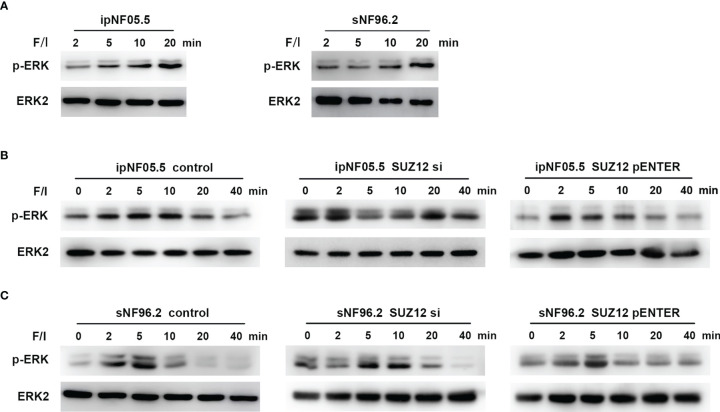
Activation of ADCY1 affects the temporal effect of ERK phosphorylation levels. **(A)** Western blot showing the activation time of P-ERK after the addition of forskolin and IBMX (F/I) in ipNF05.5 and sNF96.2. **(B)** Western blot showing changes in p-ERK expression in SUZ12 si and SUZ12 pENTER groups compared with controls in ipNF05.5. **(C)** Western blot showing changes in p-ERK expression in SUZ12 si and SUZ12 pENTER groups compared with controls in ipNF05.5. ERK2 was used as control in all figures.

Forskolin, an activator of adenylate cyclase, increases the intracellular level of cAMP (19). Additional elevation of the cAMP level can be achieved through treatment with IBMX, a nonselective phosphodiesterase inhibitor (20). Cells were stimulated with this mixture of forskolin and IBMX (i.e., F/I) for 2, 5, 10 and 20 min, and ERK was found to be activated at approximately 20 min, consistent with the manufacturer’s instructions (**Figure 5A**). F/I was added for 20 min and was then removed to observe the trend in ERK phosphorylation over time. The p-ERK level gradually increased and then decreased at 20 min in the control cells. However, the activation of ERK phosphorylation was accelerated and prolonged after transfection of siRNA, especially in ipNF05.5 cells, starting at 0 min and lasting for 40 min (**Figure 5B**). Interestingly, the level of p-ERK in the SUZ12 si group decreased at 5-10 min. However, the intensity and duration of p-ERK activation were reduced after overexpression of SUZ12. Under the same treatment conditions, SNF96.2 cells exhibited exactly the same pattern as ipNF05.5 cells (**Figure 5C**). Therefore, we confirmed that SUZ12 can affect the phosphorylation level of ERK through ADCY1.

### SUZ12 Amplifies RAS Signaling Through the cAMP/PKA/Rap1/ERK Pathway

**Figure 6:**
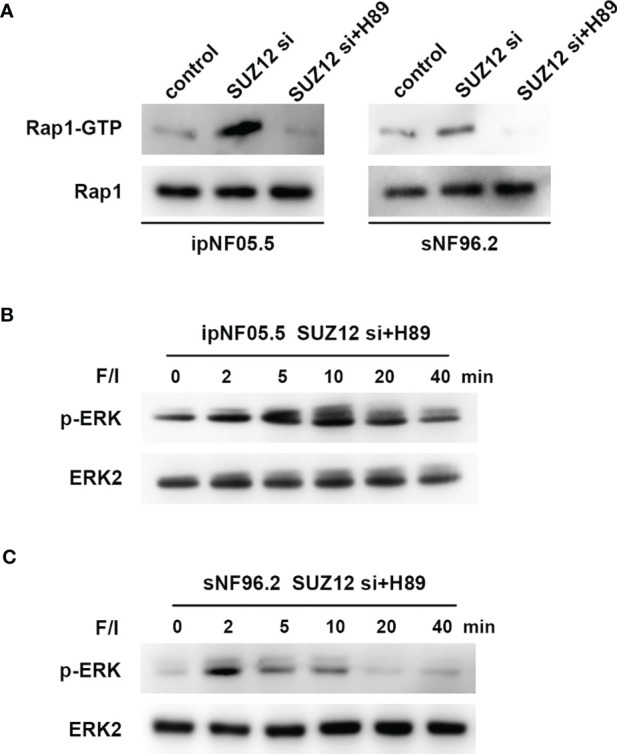
Changes in Rap1-GTP and p-ERK after the addition of PKA inhibitor (H89). **(A)** Application of pull-down method to detect the effect of knockdown of SUZ12 and addition of H89 on the expression of Rap1-GTP, the activated form of Rap1. **(B, C)** Western blot showing the effect of H89 on the level of ERK phosphorylation in the SUZ12 si group treated with F/I in both ipNF05.5 and sNF96.2, and ERK2 was used as control.

ERK activation is mediated by PKA-independent Ras proteins that cooperate with the Rap1 protein through cAMP (20). To determine whether the effect of SUZ12 is mediated by Rap1, we utilized a pulldown assay to detect the effect of SUZ12 knockdown and PKA inhibitor (H89) treatment on the level of activated Rap1 (Rap1-GTP). Rap1 activation was significantly elevated after interference with SUZ12 expression and was blocked by H89 treatment (**Figure 6A**). More importantly, the effects of SUZ12 knockdown on ERK phosphorylation were eliminated after the addition of H89 to both ipNF05.5 (**Figure 6B**) and sNF96.2 cells (**Figure 6C**) in the SUZ12 si group treated with F/I. Therefore, we confirmed that SUZ12 can amplify RAS signaling through the cAMP/PKA/Rap1/ERK pathway.

## Discussion

Clinically, NF1 patients with microdeletions have significantly more systemic complications than those without. Comprehensive genomic and clinical efforts led to the proposal that there are at least three steps required for cellular transformation during the development of MPNST, which include plexus neurofibromas, atypical neurofibromatous neoplasms of uncertain biologic potential (ANNUBP) and MPNSTs. Recurrent mutations in SUZ12 and/or EED, two key components of the polycomb repressive complex 2 (PRC2), lead to loss of tri‐methylation of histone H3 lysine 27 (H3K27me3) and de-repression of its target genes (21). Further genotypic studies revealed that SUZ12, a component of the PRC2 complex, is often codeleted. The genes targeted by PRC2 regulate cell cycle progression, stem cell self-renewal, cell fate decisions, cellular identity and tumorigenesis (22, 23).We analyzed the DEGs between SUZ12-WT and SUZ12-Mut ipNF05.5 cells and confirmed that SUZ12 regulates the transcription of numerous genes, especially those associated with the synaptic membrane, axonogenesis and DNA-binding transcription activator activity. Heart defects are significantly more common in patients with NF1 microdeletions than in patients with intragenic NF1 mutations (24). However, the type and frequency of heart defects observed in patients with NF1 microdeletions are quite heterogeneous. KEGG enrichment analysis showed that the DEGs were enriched in cardiomyopathy-related pathways in addition to the MAPK signaling pathway and the Rap1 and cAMP signaling pathway. The role of SUZ12 in cardiomyopathy is thus worthy of further investigation.

The distribution of H3K27me3 in the ADCY1 promoter region and across the genome was significantly sparser in SUZ12-Mut cells, implying that ADCY1 is regulated by SUZ12. Both the PPI network analysis results and the observed enrichment of the cAMP pathway suggest that ADCY1 may play an important role. More importantly, the survival of MPNST patients decreased with increasing ADCY1 expression. A recent study showed that SUZ12 upregulated the RAS pathway although the phosphorylation level of ERK did not change significantly after either overexpression or knockout of SUZ12 (14). Our studies supported this finding *via* GSEA in SUZ12-Mut cells.

A recent review summarized 5 articles on next-generation sequencing studies of MPNST; in these articles, the SUZ12 mutation frequency ranged from 32% to 88%, with an average of 56.1% (16). In this study, SUZ12 expression was negative in 6 of the 8 (75%) MPNST patients and was absent in all recurrent cases. We further compared the changes in the ADCY1 and p-ERK levels in SUZ12-positive and SUZ12-negative MPNST patients, and the results were consistent with the hypothesis that SUZ12 loss may affect ERK phosphorylation through ADCY1. We further transfected SUZ12 lentivirus in ipNF05.5 and sNF96.2 cell lines. It was very interesting that pNF05.5 cells, which originally expressed SUZ12 normally, did not show a significant attenuation of proliferation, in contrast to SUZ12 relative deficient cells (**Supplementary Figure S2**). And the same is true for colon cancer and GBM (14), which is worthy of further research.

**Figure 7:**
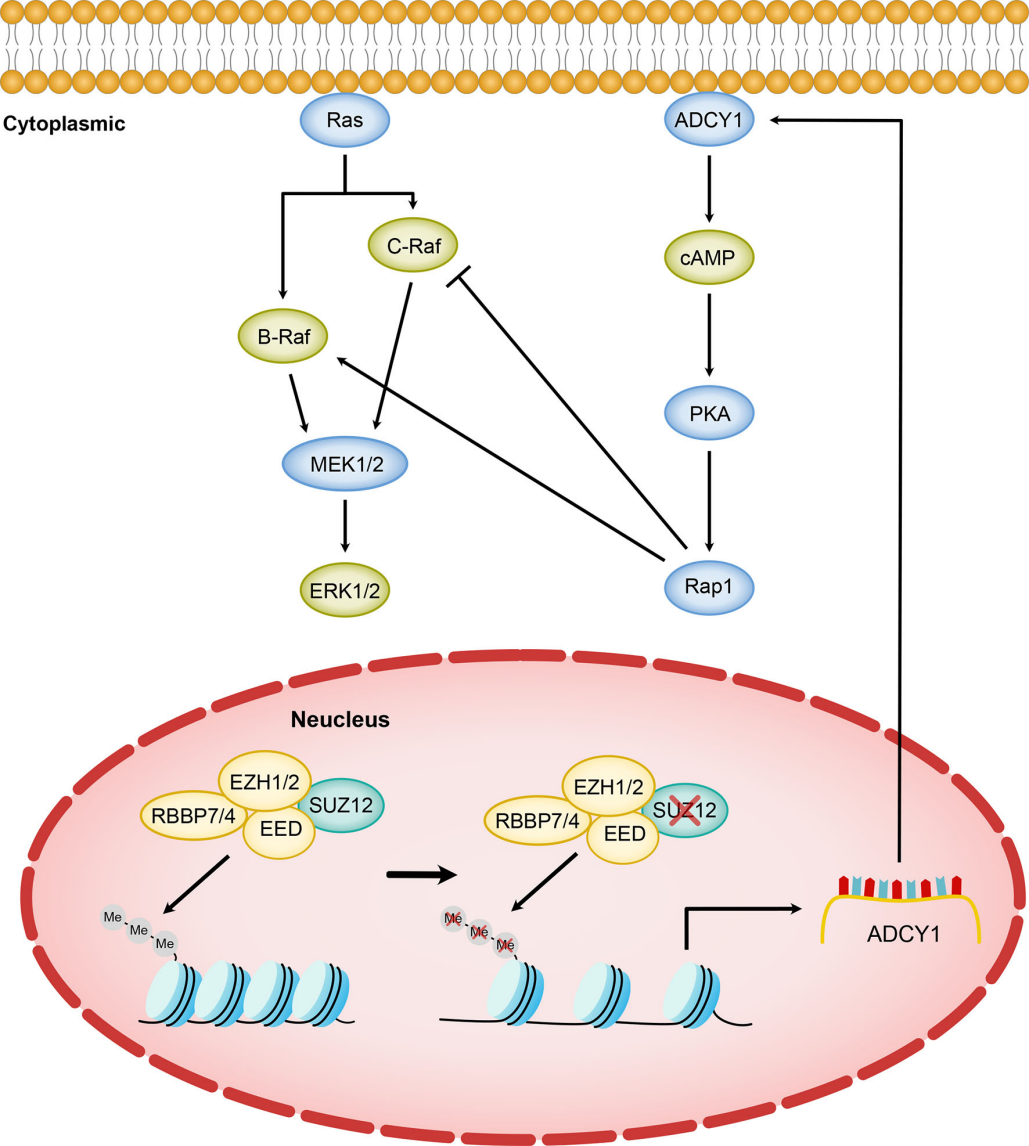
Model of SUZ12 loss amplifying the RAS/ERK pathway *via* ADCY1. Diminished transcriptional repression of ADCY1 by PRC2 after SUZ12 deletion amplifies the RAS signaling pathway through cascade activation of PKA, Rap1 and B-Raf. Different Raf isoforms may play different roles in the p-ERK activation phase.

The background in which Ras is activated in MPNST is more complex and differs in many ways from that in PNF (16). We next focused on the mechanism by which SUZ12 affects the phosphorylation of ERK. We used F/I to activate ADCY1 to produce cAMP after transfection and observed the changes in ERK phosphorylation. In both cell lines, ERK phosphorylation was accelerated and lasted significantly longer after knockdown of SUZ12, while the duration of ERK phosphorylation was decreased after overexpression of SUZ12. Studies have shown that the roles of Ras and Rap1 are distinguished by their mechanism of activation, dependence on cAMP-dependent PKA, and the magnitude and kinetics of their effects on ERKs. Ras is required for the early phase of ERK activation by cAMP and is activated independently of PKA. In contrast, Rap1-mediated activation of ERKs has a longer duration and is dependent on PKA (20, 25, 26). To confirm whether SUZ12 affects ERK phosphorylation *via* Rap1, we performed a pulldown assay and found that the change in the level of Rap1-GTP, the activated form of Rap1, was highly consistent with the change in the level of p-ERK. However, Rap1 activation was completely inhibited by the PKA inhibitor H89, and the level of p-ERK was restored to that in cells without SUZ12 siRNA transfection. Therefore, we demonstrated that the effect of SUZ12 on ERK phosphorylation is mediated through cAMP/PKA/Rap1 signaling (**Figure 7**).

However, the increasing-decreasing-increasing trend in the p-ERK level in the SUZ12 siRNA group was very interesting. Rap1 interacts with the serine/threonine kinases B-Raf and C-Raf, and active (GTP-bound) Rap1 in turn activates B-Raf, which leads to the phosphorylation cascade of MEK1/2 and ERK1/2; thus, cAMP and MAPK signaling are linked (27–29). Ras can activate all known Raf isoforms, but Rap1 activates B-Raf and inhibits C-Raf. cAMP can prevent membrane recruitment of C-Raf and hence activation of the Raf–MEK1/2–ERK1/2 cascade (30). In cells that express B-Raf and C-Raf, the effects of cAMP on ERKs may represent a balance between its effects on each Raf isoform (27). According to the above studies, we hypothesize that the trend might be due to activation of C-Raf by cAMP at the appropriate time and concentration, which in turn inhibits ERK phosphorylation (**Figure 7**). cAMP activates both B-Raf and C-Raf, which perform different functions in different cells (27, 31), and the specific mechanisms need further study.

MPNSTs are often arising from pre-existing benign plexiform neurofibromas (PN) and atypical neurofibromas (ANF), now known as ANNUBP. ANF are distinct from both PN and MPNST, representing an intermediate step in malignant transformation. Pemov and his team identified a low number of mutations and frequent deletions of CDKN2A/B (69%) and SMARCA2 (42%) in ANFs (32). And they determined that EED or SUZ12 were frequently mutated, deleted or downregulated in MPNSTs but not in ANFs. The PN-ANF transition is primarily driven by the deletion of CDKN2A/B. Further progression from ANF to MPNST likely involves broad chromosomal rearrangements and frequent inactivation of the PRC2 genes (32). The WHO 2016 Classification of CNS Tumors and the new fifth edition of the World Health Organization (WHO) Classification of Tumors of Soft Tissue and Bone in 2020 both had adopted identification of H3K27me3 as a diagnostically useful marker in MPNST (17, 33). Complete loss of H3K27me3 is a highly specific (98.7%) marker of MPNST that can distinguish MPNST from cytomorphologic mimics in FNA cell block and small biopsy specimens (34). Cleven et al. found that malignant peripheral nerve sheath tumors with loss of H3K27 tri-methylation showed inferior survival compared with malignant peripheral nerve sheath tumors with intact H3K27 tri-methylation (35). De Raedt et al. demonstrated that SUZ12 loss promotes an epigenetic switch from H3K37Me3 to H3K27Ac, conferring sensitivity to BRD4 inhibitor-based combination therapies. BRD4 inhibitors alone may not be effective in these tumors, but should be evaluated in combination with MEK inhibitors (14). However, the MPNST genes are frequently mutated, and the current studies are clearly not enough.

In conclusion, our study sought to determine the mechanism by which SUZ12 enhances the RAS/ERK pathway, as shown, and provided insights into the effects on the temporal characteristics of ERK phosphorylation mediated by SUZ12 through ADCY1. Furthermore, it may provide a new basis for MEK inhibitor combination chemotherapy.

## Data Availability Statement

The raw data supporting the conclusions of this article will be made available by the authors, without undue reservation.

## Ethics Statement

The studies involving human participants were reviewed and approved by Beijing Tiantan Hospital Ethics Committee of Capital Medical University. Written informed consent to participate in this study was provided by the participants’ legal guardian/next of kin.

## Author Contributions

WL, CH, XZ, DL, and SL contributed to the conception and design of the study. WL, XZ, BW, ZL, SS, and CG contributed to the experimental operations. WL, CH, BW, ZL, DL, and SL contributed to the acquisition and analysis of data. WL, CH, XZ, BW, ZL, DL, and SL contributed to the drafting of the text and the preparation of the figures. All authors contributed to the article and approved the submitted version.

## Funding

This work was supported by grants from Platform Construction of Basic Research and Clinical Translation of Nervous System Injury (PXM2021_026280_000006, Beijing Municipal Health Commission), and the Institut pour la Recherche sur la Moelle Epinière et l’Encéphale (IRME, Paris, France).

## Conflict of Interest

The authors declare that the research was conducted in the absence of any commercial or financial relationships that could be construed as a potential conflict of interest.

## Publisher’s Note

All claims expressed in this article are solely those of the authors and do not necessarily represent those of their affiliated organizations, or those of the publisher, the editors and the reviewers. Any product that may be evaluated in this article, or claim that may be made by its manufacturer, is not guaranteed or endorsed by the publisher.

## Supplementary Material

Supplementary Figure S1 Overall survivals of 4 hub gene were performed using COX survival analysis. High expression of ADCY1 showed worse survival **(A)**, while the other three genes including ADRA2C, BDKRB1 and BDKRB2 had no significant difference in the survival rate in MPNST **(B–D)**.

Supplementary Figure S2 Effects of overexpression of SUZ12 on proliferation of sNF96.2 and ipNF05.5 cell lines. sNF96.2 and ipNF05.5 cell lines were transfected with SUZ12 lentivirus, and proliferation ability was tested by CCK-8 kit. The results showed that the proliferation of sNF96.2 cells was significantly reduced **(A)**, but ipNF05.5 was not **(B)**. (Two-way ANOVA, *P < 0.05).
