# Supplementary figures and images for: SUZ12 Loss Amplifies the Ras/ERK Pathway by Activating Adenylate Cyclase 1 in NF1-Associated Neurofibromas
Source: Front Oncol. 2021 Oct 6;11:738300. doi: 10.3389/fonc.2021.738300 (PMC8526866; doi:10.3389/fonc.2021.738300)

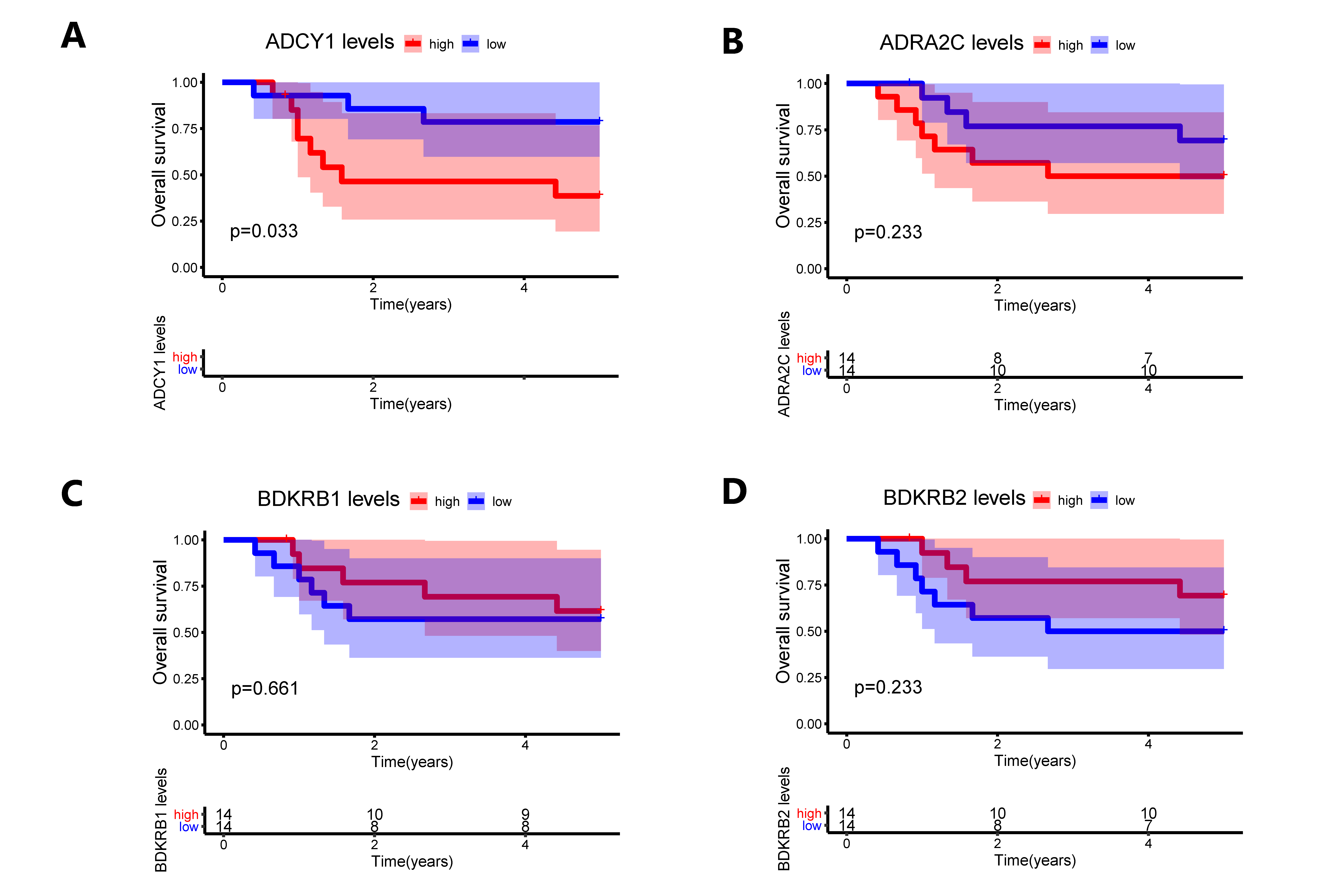

Supplement: Supplementary Figure S1 — Overall survivals of 4 hub gene were performed using COX survival analysis. High expression of ADCY1 showed worse survival (A), while the other three genes including ADRA2C, BDKRB1 and BDKRB2 had no significant difference in the survival rate in MPNST (B–D). [file Image_1.tif]

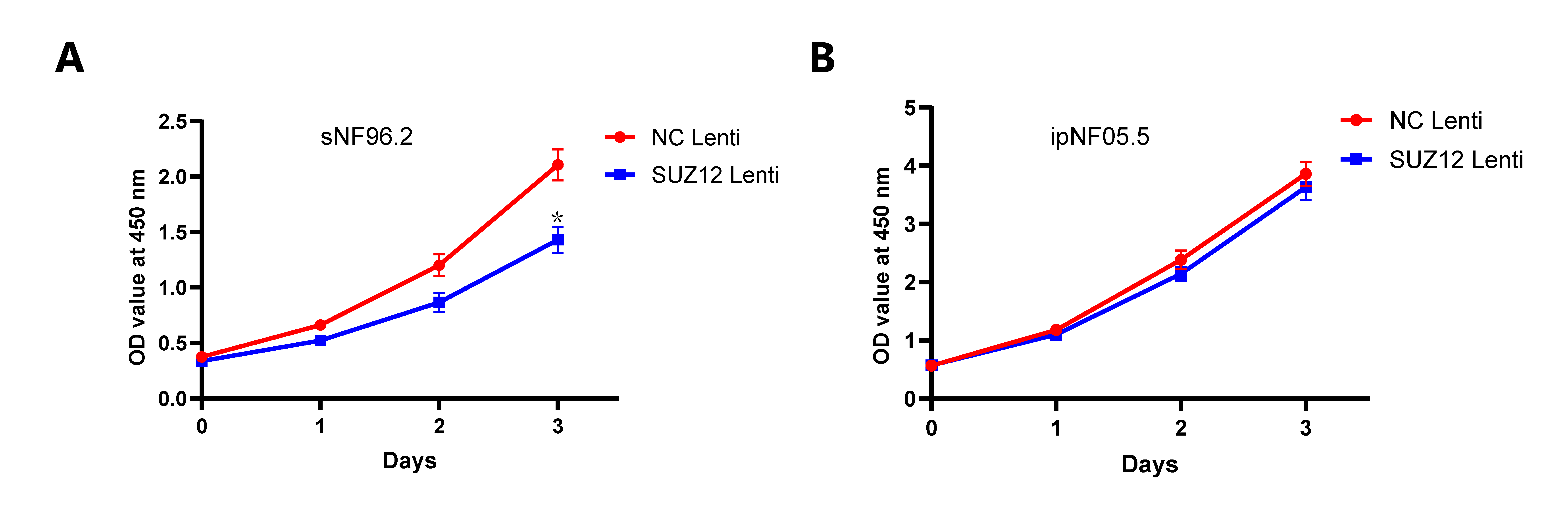

Supplement: Supplementary Figure S2 — Effects of overexpression of SUZ12 on proliferation of sNF96.2 and ipNF05.5 cell lines. sNF96.2 and ipNF05.5 cell lines were transfected with SUZ12 lentivirus, and proliferation ability was tested by CCK-8 kit. The results showed that the proliferation of sNF96.2 cells was significantly reduced (A), but ipNF05.5 was not (B). (Two-way ANOVA, *P < 0.05). [file Image_2.tif]
