# Supplementary material for: SUZ12 Loss Amplifies the Ras/ERK Pathway by Activating Adenylate Cyclase 1 in NF1-Associated Neurofibromas
Source: Front Oncol. 2021 Oct 6;11:738300. doi: 10.3389/fonc.2021.738300 (PMC8526866; doi:10.3389/fonc.2021.738300)
Supplement: Supplementary file 3 [file Table_1.doc]

Supplementary Table 1 All differentially expressed genes (DEGs) with SUZ12 mutation in ipNF05.5 cell

Down

*ABCB5 GAS7 PLEKHB1 ROS1 CELF2 ARHGEF5 COL11A1 SPAG4 NGFR ERBB3 CDH19 MPP4 FOLH1 FER1L4 SUSD2 SOX10 PNPLA3 CHRDL1 ASB9 LMF1 SFRP1 ITGB8 PTPRZ1 RARRES2 CA9 FOLR3 AGXT2 CNTN3 ATP10B PPL ZSCAN18 FABP3 COL10A1 PLP1 CHN1 ITGB4 AMPD3 EHF MYPN CHRNA1 PADI3 RGS16 ADAMTSL4 SELENBP1 WNT9A ST8SIA6 ADIRF PLEKHS1 PAMR1 KIAA1755 ABI3BP GPR78 MAPK13 FCER1G S100B ZNF208 FGFR4 LAPTM5 MEGF6 B3GALT2 FRZB DNALI1 FABP7 SERPINB7 PLEKHA7 FN3K ACOX2 CAVIN2 FSTL5 VXN FAXDC2 PDZK1 C15orf54 ADAMTSL1 CA8 PLCXD1 CCBE1 ACSM5 PSG9 MACC1 ZNF730 TRPV2 SLC38A3 SERPINA5 LCTL ZBED6CL S100A4 ZNF429 ACSL5 DPP4 TUBA3C MAGEE1 MOG PSG5 RGL3 UCA1 TSSC2 C4B LINC01204 FOXD3-AS1 LINC01198 PSG1 PAGE2 LINC01505 ZNF737 LINC00886 PSG4 C4A LOC110384692 LOC643201 SELENOP LOC105369187 PCDHGA12 LINC01419 LOC100130111 TRIM6-TRIM34 LINC00639 PCDHGB3*

*PCDHGA4 LINC02864 GJA5 LINC01224 PCDHGB5 RAB7B LOC653653*

Up

*TNMD CYP26B1 SLC7A2 MEOX1 CRLF1 ARHGAP44 PROM1 CEACAM21 MMP25 PAX7 FMO1 SYT7 ALOX5 CYP24A1 RRAGD HSD17B6 KCNG1 APBA2 TLL1 ZIC2 LCP2 LMO3 TNFRSF9 LAMC3 ALX4 PTPRN CYFIP2 PKP2 PTPRU PRDM6 EYA2 SNCAIP NTN1 SNAP91 PRKCQ TLE2 NEO1 SYT1 PDZD4 FGFR3 REEP1 RASGRP2 PITX1 SDK2 GAL MGAT4 ADGRL1 CRMP1 MOV10L1 NOTCH3 PPP2R2C TEAD2 NUAK1 ADD2 CACNG4 PAX2 PAG1 GPC4 RAP1GAP FBLN1 ITGA8 LAMP3 COL5A3 PCDHB4 ABCB1 SNX10 FAT2 GNAO1 TFAP2C DOCK3 EBF4 CPXM1 TBX5 CHRD NRCAM PITPNM3 JPH4 PHGDH PTGS1 TLL2 CRTAC1 MACROH2A2 EFNA2 PALM CECR2 GRK3 TTC28 SEPTIN3 COCH TRIM9 SLC8A3 BDKRB1 MMP9 R3HDML NFATC2 SALL4 BMP7 NKAIN4 EEF1A2 RASSF2 RSPO4 HCK CDH20 LIPG LAMA1 GPR50 NALCN HTR2A SGCG FLT1 ZNF423 SMPD3 FOXF1 SYT17 CEMIP HOMER2 FAM189A1 PDGFRL EYA1 JPH1 ESRP1 STMN2 NOVA2 OLFM2 ATP1A3 CACNG7 TMEM59L LSR HOXA2 HOXA3 HOXA5 CPVL CHN2 EPHB6 LHX2 CNTNAP3 PRUNE2 ELAVL2 PIP5K1B SH3GL2 GATA3 PALD1 UNC5B DKK1 RNF43 EFNB3 ODAM CRACD PPARGC1A CALCA KRT18 PPM1H MGP SLC38A1 TBC1D30 ULBP1 BACH2 SOBP PDE10A LINC00473 MDFI ENPP5PRS S16 TBX18 PCDHB2 PCDHB3 PCDHB5 PCDHB6 CDH6 NPR3 SLC12A7 HRH2 VIPR1 ADAM23 IGFBP5 EFHD1 ST3GAL5 IL18R1 SLC9A2 PAPPA2 NCF2 MYCL ZP4 RIMS3 TNFSF4 PLPPR4 PROX1 CNR1 CCND2 GALNT12 ONECUT2 MSX2 MOB3B PLEKHG1 MYCT1 TNFSF18 ENOX1 CLU NPPB MAPK8IP1 PAEP INHBA EGR2 ITIH5 PDE1B HOXC13 TNFAIP6 KCNJ2 PREX1 TREM1 TMEM255A BMP4 IL1B NKX2-2 LAMP5 TMEM74B GPR42 BCL11B TNFRSF19 RASL11B ADM2 MGAT3 KRT17 HOXD10 HOXD13 ISLR EGLN3 KCNA6 GALNT8 CRACR2A CNN1 KIF1A CACNG6 UNC13A KLHDC7B ASS1 DUSP9 LRP3 GFPT2 ANO1 BARX1 RAI2 PPARG EMILIN2 ATP8A2 CHRM3 DCLK1 CCNA1 POSTN BEX2 BEX1 PDZD2 TMTC1 TTC9 TSPAN2 CYP2J2 DSC3 NREP FAM189A2 HRK ANKRD6 LMO2 AGAP2 NEK3 NKX2-1 ZFHX2 TM6SF1 SCN7A GATA4 KLF4 TMOD1 LMX1B ENPP2 SLC22A23 TUBB2B LRRC32 SULF1 SORL1 FXYD6 ARHGAP20 ITGA11 STRA6 SEMA6D SLC44A5 CYP1B1 CH25H RBP4 CILP HCN4 FRAS1 B4GALNT3 SLC38A4 PPFIA2 TMEM132B SLAIN1 ZIC5 GRTP1 SSTR1 RTN1 SYT16 FBLN5 FGF7 PCSK6 NTRK3 SH3GL3 ITGAX NKD1 MYOCD KSR1 GNAL ADCYAP1 SLC14A1 SLC13A5 RNF165 CACNA1A ADAMTS10 CACNG8 ILDR2 CRABP2 SYT14 SUSD4 KCNN3 FLG AFF3 NYAP2 ACKR3 ITGA9 IL17RD TMEM108 STXBP5L SFRP2 ADAMTS16 OTULINL TSLP MEGF10 TENM2 KCNMB1 CLVS2 RSPO3 VIP SLC22A3 IGFBP3 IGSF1 ARHGAP36 CSGALNACT1 SYBU CDKN2B SHC3 GBGT1 HMCN2 HTR7 MKX MPP7 ADRA2A MAT1A CCDC3 ANO4 EDNRA RNF144A TDO2 ASTN1 PDE3B GUCY1A2 XRCC4 MERTK ADGRF4 PLEKHG4B JPH3 RETREG1 C4orf19 CNTNAP3B WNT7A FGD5 PIEZO2 EPHB1 DKK2 DEPTOR PPARGC1B RAB39B TSPAN7 GDF6 HKDC1 CACNA1D KIT CACNA2D3 PWWP3B ERG TSPAN18 DGKI COLEC12 CLIC6 IGF2BP1 SIM2 TCHH FAM131B KALRN ABCG1 TFF3 TFF2 TMPRSS3 CBS ICOSLG MPZL3 DMKN GRASP RNASEK-C17orf49 SHANK2 TAL1 DRAXIN PDPN ALPL WNT4 DIRAS3 NFIA VCAM1 CADM3 VANGL2 ACTG2 VSNL1 EN1 SPATA18 SPRR2D CLDN1 FCRL4 CADPS CDS1 GUCY1A1 NPY1R HHIP ELOVL7 F2RL1 ESM1 EGFLAM EBF1 TMEM200A TBX20 ZNF704 TAGAP FNDC1 SOX17 ADCY1 TMEM74 SVEP1 PGAP4 SLC16A9 EML5 TMEM63C OTX2 JCAD NDRG2 BTNL9 ADAMTS15 SCN3B BEAN1 BMERB1 RBPMS2 ELFN2 MS4A8 ISLR2 MYO5B LPO LAIR1 LAIR2 SEMA6B NXN BDKRB2 COL3A1 GFRA2 LDLRAD4 LRP1B NPNT NSG1 ACTBL2 ROR2 PARM1 ARMC4 EFNA1 STK32A ELSPBP1 COL22A1 SDC2 ZNF280A LINGO1 PCDH7 EMX2 SEMA3E LRRN2 LONRF2 GPR37 HTRA3 GPR27 OSCAR HS6ST2 FAM241B CDK5R2 MAP6 GATM SCG2 CALB2 FRMD3 RCAN2 RASGRP1 SH3RF3 ADCY5 SYT12 STOX2 NDNF JUP CBX2 SELP CHRNA9 CMKLR1 SLCO2A1 C3orf36 BRSK2 FZD4 BTC FUT1 SPSB4 ABO PPM1E TP53I11 PCSK1 LPL SUGCT ETV4 CALCB PRR15 SCN4B FBXO39 IRX3 NHLH2 PGBD5 C12orf54 TRIML2 HTR1F CDH4 WSCD1 HLA-DQB1 GPC5 HTR1D C3orf80 SHISA2 S1PR5 GREM2 RPH3AL CHRM2 ZNF467 FDCSP TNFSF15 CACNB4 TSHZ2 BGN PLCB1 PAPPA GRID1 CAMK1D NKX2-5 BEGAIN CSMD1 GPR1 OPCML KREMEN1 KCTD16 B3GALT5 SCN5A ST6GALNAC3 ADRA2C PTP4A3 BEX5 LPAR5 PDE4B ZBTB7C DRD1 TCEAL2 SORCS2 ANKS1B GPRIN3 ROR1 SV2B PRKG1 PBX1 KRT79 NTF3 FFAR3 EDARADD KRT3 PRR5 KRT16 CCK FPR3 TLR5 AMTN LIN28B TMEM215 COL4A5 JAKMIP3 C22orf34 COL25A1 HMX3 IGFL3 BEND4 RELN GAGE2B GAGE2A GAGE8 ANKRD34B NCR1 HLA-DRB1 GIMAP5 STK31 NTNG2 SULF2 MMP1 WNK3 DAPK1 KIAA1211L SLC6A17 DCHS2 OGDHL MFAP5 OCLN PLCG2 ELOVL2 ZNF334 CARD11 HLA-DRB5 ATL1 PPP1R14C RYR3 TOX POU3F3 RORB SAMD5 NHSL2 FLJ44635 COL15A1 IGFL2 IGFL4 AARD INSYN1 ATP10A COL6A6 TMEM200C DIO2 ACKR1 S1PR3 C9orf47 HBE1 MEG3 PLXNA4 RFPL4A LINC01133 ZNF469 LINC00937 LINC01429 RFPL4AL1 HLA-DRB6 SFTA3 EMX2OS ZBED9 RAB6D LOC105369306 LINC01239 ZNF853 PRKCQ-AS1 RTL5 PCDHAC2 RPLP0P2 FRG1-DT CEBPA LOC283731 LINC01411 HOXC13-AS CTD-2297D10.2 HOXA10 TBX5-AS1 PCDHB17P SALL3 CASC18 DIO3OS LOC100506388 MYZAP ZNF488 CEBPA-DT GABRQ FENDRR LOC93429 IGFL2-AS1 ADGRE4P TRABD2B LINC01480 CD24 DOC2B GAGE13 KRTAP7-1 ZNF280B GAGE12B FLJ16779 DACH1 NEFL FAM95C LOC339166*
